# Supplementary material for: Control of neuronal excitation–inhibition balance by BMP–SMAD1 signalling
Source: Nature. 2024 Apr 17;629(8011):402–9. doi: 10.1038/s41586-024-07317-z (PMC11078759; doi:10.1038/s41586-024-07317-z)
Supplement: Supplementary file 1 — This file contains additional Methods, references and Supplementary Fig. 1. [file 41586_2024_7317_MOESM1_ESM.pdf]

nature portfolio

https://doi.org/10.1038/s41586-024-07317-z

## Supplementary information

# Control of neuronal excitation–inhibition balance by BMP–SMAD1 signalling

In the format provided by the authors and unedited

## Additional Methods

**Recombinant DNA and viral vectors.** The following plasmids were obtained from Addgene: pAAV-hSyn-DIO-hM4D(Gi)-mCherry (RRID:Addgene_44362, gift from Bryan Roth), p4XBRE-SBE-SV40-GL3 (RRID:Addgene_67811, gift from Ron Prywes), pGL4.10/RSV_SF3B3-miniXon_Luciferase (RRID:Addgene_174660, gift from Beverly L. Davidson), FingR-PSD-95-eGFP-CCR5TC and FingR-Gephyrin-eGFP-CCR5TC (RRID:Addgene_46295 and RRID:Addgene_46296, gift from Donald Arnold), Xph20-eGFP-CCR5TC (RRID:Addgene_135530, gift from Daniel Choquet), pAAV-CAG-mGreenLantern (RRID:Addgene_164469, gift from Gregory Petsko), pAAV-S5E2-dTom-nlsdTom (RRID:Addgene_135630, gift from Jordane Dimidschstein).

pAAV-4xBRE-SV40-$X^{on}$-GFP and pAAV-4XBRE-SV40-DIO-$X^{on}$-GFP were generated by combining 4XBRE-SV40 promoter elements, the $X^{on}$ mini-cassette, and eGFP sequences, flanked by loxP and lox2272 sites in the case of double floxed inverted orientation (DIO). In pAAV-hSyn-DIO-$X^{on}$-GFP the 4XBRE-SV40 sequence was replaced by the human Synapsin (hSyn) promoter. pAAV-Tet-DIO-hSyn-HAcaBMPR1A-rtTA3 and pAAV-Tet-DIO-hSyn-HAcaCD4-rtTA3 were generated by combining human BMPR1A sequence with Q233D mutation or human CD4 sequence, hSyn promoter, Tet-operon casette, doxycycline- inducible rtTA3 transcriptional activator, HA tag sequence, and flanked by lox66 and lox71 sites for double floxed inverted orientation (DIO).

For conditional viral expression of intrabodies in PV interneurons, FingR or Xph20 coding sequences were introduced into pAAV vectors in double-floxed inverted orientation under control of the hSyn promoter combined with the CCR5 zinc finger interaction site. For expression of FingR-PSD-95 binder selectively but in a cre-independent manner in PV interneurons, S5E2 enhancer sequence was introduced into pAAV vectors upstream of FingR coding sequence combined with CCR5 zinc finger interaction site. For both enhanced conditional and S5E2-PSD-95 binders, eGFP was replaced by mGreenLantern coding sequence.

Viral supernatants were produced by co-transfection of HEK293T cells grown on 15cm dishes using calcium phosphate transfection of 70µg of AAV helper plasmid (Rep/Cap, Serotype 9), 200µg of AAV pHGTI-adeno1 (Plasmid factory) and 70µg of AAV vector plasmid carrying the cDNAs to be expressed. 45-60h after transfection, medium containing viral particles was harvested and purified using the Iodixanol purification method. Viral preparations were concentrated in Millipore Amicon 100K columns at 4°C. Virus samples were suspended in PBS, frozen in aliquots and stored at -80°C. Viral titers were determined by qPCR and were $>10^{13}$ particles/mL.

**Primary neuron culture.** Cortical cultures were prepared from E16.5 mouse embryos or newborn (P0) mice. Neocortices were digested by addition of papain (145 units in 7 ml, Worthington Biochemical #LK003176) for 30 min at 37°C and then mechanically dissociated. Cells were maintained in neurobasal medium (Gibco #21103) containing 2% B27 supplement (Gibco #17504-044), 2mM Glutamax (Gibco #35050-038), and 1% penicillin/streptomycin (Gibco, #15140122) at 37°C / 5% $CO_2$. For neuronal network activity stimulation experiments, cortical cultures were treated for 6 hours at day *in vitro* 14 (DIV14) with 25 mM KCl or with 20 µM bicuculline (Tocris #0130). For immunohistochemistry, ChiP-seq or RNA-seq experiments, DIV14

cultures were stimulated with 20 ng/ml human recombinant BMP2 (R&D systems, #355-BM-050), recombinant mouse Noggin (R&D Systems, 1967-NG-025), or vehicle. For neuron-specific *Smad1* loss of function experiments, cortical cultures from P0 *Smad1*$^{fl/fl}$ mice were infected at DIV9 with AAV9-hSyn-iCre virus at a multiplicity of infection of 20,000 or with a AAV9-hSyn-eGFP virus as negative control.

**RNA isolation and reverse transcription.** Mice were anesthetized with isoflurane (Baxter AG, Vienna, Austria) and brain was taken out into ice-cold PBS solution. Injected area was then dissected under Binocular Stereo Microscope (Olympus #MVX10) by using red fluorescence signal from mCherry expression. Dissected tissue was harvested in Trizol reagent (Sigma, T9424). Total RNAs were isolated and DNase treated on columns (RNeasy Micro kit, Qiagen, 74004) following the manufacturer's instructions. The cDNA libraries were built using between 100 and 200ng RNA reverse transcribed with ImPromII Reverse Transcriptase (Promega, #M314A), RNasin™ Plus RNase Inhibitor (Promega, #N261B), ImPromII 5X Reaction Buffer (Promega, #M289A), dNTPs (Sigma, D7295) and oligo(dT)$_{15}$ primer (Promega, C1101). Primary cortical cultures were washed 1x with PBS and lysed using Trizol reagent (Sigma, T9424) and followed by total RNA isolation and cDNA library preparation as described above.

Real-time quantitative PCRs were performed either with FastStart Universal SYBR GreenMaster (Roche, 04-913-850-001) or FastStart Universal Probe Master (Roche, 04-913-195-7001). PCRs were carried out in a StepOnePlus qPCR system (Applied Biosystems) and were analyzed with the StepOne software. Gene expression assays were used either with FastStart Universal SYBR GreenMaster (Roche, 04-913-850-001) or TaqMan Master Mix (Applied Biosystems) and comparative $C_T$ method. The mRNA levels were normalized to housekeeping β-actin mRNA or to Gapdh mRNA. For each assay, two to three technical replicates were performed and the mean was calculated.

Commercially available gene expression assays for Fos (Mm00487425_m1), Bdnf (Mm04230607_s1), *Id1 (Mm00775963_g1), Id3* (Mm01188138_g1)*, Smad6* (Mm00484738_m1)*, Smad7* (Mm00484742_m1)*, ActB (Mm00607939_s1)* (Mm.PT.58.13518911), *Bmp2* (Mm01340178_m1), *Bmp4* (Mm00432087_m1), *Bmp6* (Mm01332882_m1), *Bmp7* (Mm00432102_m1), *Gapdh* (Mm99999915_g1) were from ThermoFisher.

Custom primer sequences were as follows:

| Primer Name | Sequence |
|---|---|
| Brevican-Exon11-Fwd | 5'-CAT CGA GGG TGA CTT CTT GT-3' |
| Brevican-Exon12-Rev | 5'-ACC ATG ACC ACA CAG TTC TC-3' |
| Id3-Exon1-Fwd | 5'-GCA GCG TGT CAT AGA CTA CAT C-3' |
| Id3-Exon2-Rev | 5'-GTC CTT GGA GAT CAC AAG TTC C-3' |
| Grin3a-Exon7-Fwd | 5'-CTG CTG CTA CCA CGA ATC AA-3' |
| Grin3a-Exon8-Rev | 5'-TCT TGG AAC ATG GCT GCT T-3' |
| ActB-Exon5-Fwd | 5'-AGA TTA CTG CTC TGG CTC CTA-3' |
| ActB-Exon6-Rev | 5'-CTG CTT GCT GAT CCA CAT CT-3' |

**Western blotting.** Primary cortical cells were lysed in 50 mM Tris HCl pH 7.5, 150 mM NaCl, 10% Glycerol, protease inhibitor Roche Complete™ mini, 1% Triton X-100). Transfected HEK293T cells were lysed in 50 mM Tris HCl, 150 mM NaCl, 1.0% (v/v) NP-40, 0.5% (w/v) Sodium Deoxycholate, 1.0 mM EDTA, 0.1% (w/v) SDS). Lysates were centrifuged for 10 minutes at 16'000 g at 4°C and solubilized proteins were analyzed by polyacrylamide gel electrophoresis on 4%-20% gradient gels (BioRad, 4561093) followed by transfer onto nitrocellulose membrane. For enhanced chemiluminescence detection, WesternBright ECL kit (Advansta #K 12045-D20) and WesternBright Quantum (Advansta #K-12042-D20) were used. Signals were acquired using an image analyzer (Bio-Rad, ChemiDoc MP Imaging System and Li-Cor, Odyssey).

**Fluorescent *in situ* hybridization.** Multiplex fluorescent *in situ* hybridization (FiSH) was performed using the RNAScope Fluorescent Multiplex Kit (Advanced Cell Diagnostics, ACD). Mouse brains were snap frozen in liquid nitrogen and 18 µm coronal sections were cut between Bregma -1.43 and -2.15 (including barrel cortex and dorsal hippocampus) on a cryostat. Sections were fixed at 4°C overnight with 4% paraformaldehyde in 100mM phosphate buffered saline, pH 7.4. The procedure followed the manufacturers' instructions. For *in vitro* FiSH experiments, cortical cultures were plated onto glass coverslips. At DIV12, cells were fixed with 4% PFA for 15 minutes. The following probes were used: *Bmp2* (ACD #406661), *Camk2* (ACD #411851) and *Pvalb* (ACD #421931). Images were acquired with an upright LSM700 confocal microscope (Zeiss) using 40x/1.3 or 63x/1.4 Apochromat objectives. Cell types were identified based on the presence of the corresponding marker transcript. A region of interest (ROI) was drawn to define the area of the cell and dots in the ROI were manually counted. The number of dots in the ROI were then normalized to the cell area. Image acquisition and counting was done blinded to the experimental condition.

**Electrophysiology.** Cortical slice preparation from adolescent (P26-P28) or adult mice (P56-72) was adapted from previously described protocols [68]. Briefly, animals were anesthetized with isoflurane (Baxter AG, Vienna, Austria). Parasaggital slices of 300 µm were cut with a vibratome (VT1200S, Leica) in ice-cold oxygenated (95% $O_2$/5% $CO_2$) NMDG solution (93 mM NMDG, 93 mM HCl, 2.5 mM KCl, 1.2 mM $NaH_2PO_4$, 30 mM $NaHCO_3$, 20 mM HEPES, 25 mM glucose, 5 mM sodium ascorbate, 2 mM Thiourea, 3 mM sodium pyruvate, 12 mM N-acetyl L-cysteine, 10mM $MgSO_4$ and 0.5 mM $CaCl_2$, pH 7.35). Slices were kept at 33.0 ± 1 °C in oxygenated NMDG solution for 12 minutes and then transferred to artificial cerebrospinal fluid (aCSF; 125 mM NaCl, 2.5 mM KCl, 1.25 mM $NaH_2PO_4$, 24 mM $NaHCO_3$, Na-Ascorbate (5 mM), 12.5 mM glucose, 1 mM $MgCl_2$ and 2 mM $CaCl_2$, pH 7.4) and kept at room temperature for at least 1 h before starting the recordings. During the recording sessions the slices were held in a custom chamber heated to 33.0 ± 1 °C with oxygenated aCSF perfusion.

Whole-cell patch-clamp recordings from layer2/3 PV interneurons of the barrel cortex were performed in voltage or current clamp mode using pClamp11 software with Multiclamp 700B amplifier (Molecular Devices, Sunnyvale, CA) and under visualization in an upright microscope (Olympus) equipped with gradient contrast infrared visualization (Luigs and Neumann) using a 60× objective. For all experiments, data was digitized by Digidata 1440a (Molecular Devices) at

10 kHz and filtered at 1 kHz. For mEPSC and mIPSC recordings, patch pipettes (3–6 MΩ) were pulled with Sutter P-1000 micropipette puller (Sutter Instruments) and were filled with the following intracellular solution: 130 mM $CsMeSO_3$, 8 mM NaCl, 4 mM Mg-ATP, 0.3 mM Na-GTP, 0.5 mM EGTA, 10 mM HEPES, 5 mM QX314. For excitability measurements, intracellular solution composition is 142 mM K-gluconate, 10 mM HEPES, 1 mM EGTA, 2.5 mM $MgCl_2$, 4 mM Mg-ATP, 0.3 mM Na-GTP, 10 mM Na-phosphocreatine. mEPSCs and mIPSCs were recorded in the presence of 1 μM tetrodotoxin (TTX). To isolate both mEPSCs and mIPSCs from the same cell, mEPSCs were recorded at -70 mV holding potential and mIPSCs at 0 mV holding potential. Cells with >20% change in the series resistance were excluded from the analysis. mEPSCs and mIPSCs were analyzed using a template-matching algorithm implemented in Clampfit 10 (Molecular Devices). Automatically detected events were visually controlled and false positive events were deleted. Amplitude and frequencies were then manually analyzed. Input resistance, membrane time constant and capacitance were also calculated in Clampfit 10. Action potentials of PV interneurons were automatically detected with Neuromatic [69] and analyzed using a custom-made script written for this project in Igor Pro 8 software (WaveMetrics).

Cortical slice preparations and excitability measurements from layer 2/3 PV interneurons of P26-P30 mice were performed as described above with the following modifications: Slices were cut in sucrose-based solution: 75 mM sucrose, 87 mM NaCl, 25 mM $NaHCO_3$, 2.5 mM KCl, 1.25 mM $NaH_2PO_4$, 0.5 mM $CaCl_2$, 7 mM $MgCl_2$ and 10 mM glucose. Slices were immediately transferred to a storage chamber containing artificial cerebral spinal fluid (aCSF) containing: 125 mM NaCl, 25 mM $NaHCO_3$, 2.5 mM KCl, 1.25 mM $NaH_2PO_4$, 2 mM $MgCl_2$, 2.5 mM $CaCl_2$ and 11mM glucose, pH 7.4, constantly bubbled with 95% $O_2$ and 5% $CO_2$; 315-320 mOsm. Slices were maintained at 35°C in aCSF for 60 min and then kept at room temperature before their transfer to the recording chamber. During the recordings, the slices were continuously perfused with aCSF at 35.0 ± 2.0°C throughout the experiments. Neuronal activity of layer 2/3 PV interneurons was recorded with borosilicate glass pipettes (4-6 MΩ) filled with an intracellular solution containing: 125 mM K-gluconate, 20 mM KCl, 10 mM HEPES, 10 mM EGTA, 2 mM $MgCl_2$, 2 mM $Na_2ATP$, 1 mM $Na_2$-phosphocreatine, 0.3 mM $Na_3GTP$ and 0.2% biocytin. Passive membrane properties and detection of action potentials were measured by using a custom-made script in Igor Pro 8 software (WaveMetrics).

**Open field test.** Behavioral testing was done with males and females which were aged between 10 and 16 weeks. Mice were handled for at least 3 days before the test and acclimatized to the testing room for at least an hour before starting the experimentation. Mice were placed in the center of a rectangular OFT box (30 cm in width and 45 cm in length, with 30-cm-tall walls) for 15 minutes. Videos were recorded with a downward-facing camera from above with ANY-maze at a rate of up to 30 Hz. Distance (as cm) was extracted from ANY-MAZE and velocity (cm/min) was calculated.

**References**


68 Jiang, X. *et al.* Principles of connectivity among morphologically defined cell types in adult neocortex. *Science,* **350**, aac9462 (2015). https://doi.org/10.1126/science.aac9462

69 Rothman, J. S. & Silver, R. A. NeuroMatic: An Integrated Open-Source Software Toolkit for Acquisition, Analysis and Simulation of Electrophysiological Data. *Front Neuroinform* **12**, 14 (2018). https://doi.org/10.3389/fninf.2018.00014

Supplementary Figure 1 (uncropped western blots)

## Extended Data Figure 1e

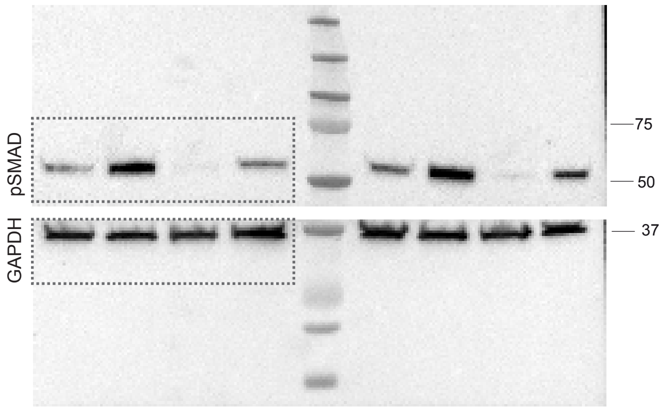


## Extended Data Figure 1g

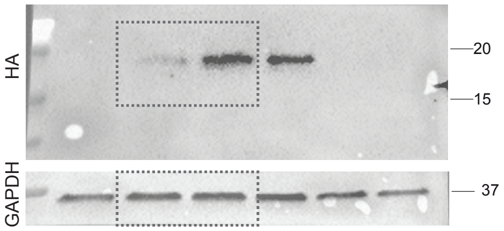


## Extended Data Figure 2c

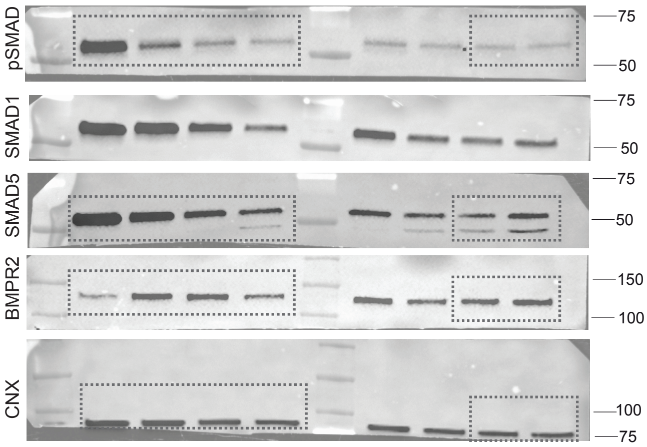


## Extended Data Figure 4c

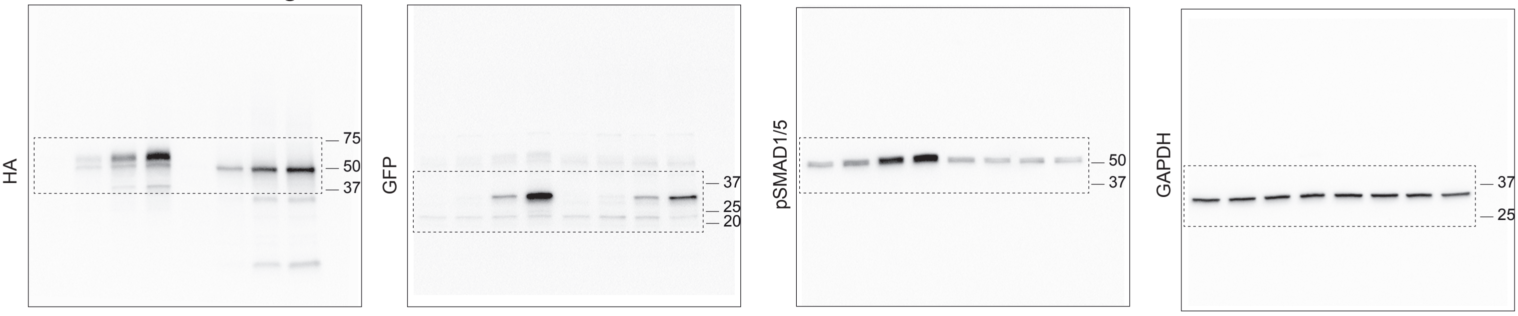


## Extended Data Figure 5e

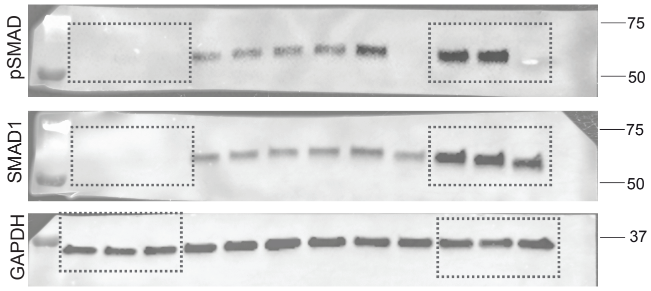


panel was reflected
