# Supplementary material for: Control of neuronal excitation–inhibition balance by BMP–SMAD1 signalling
Source: Nature. 2024 Apr 17;629(8011):402–9. doi: 10.1038/s41586-024-07317-z (PMC11078759; doi:10.1038/s41586-024-07317-z)
Supplement: Supplementary file 2 — Reporting Summary [file 41586_2024_7317_MOESM2_ESM.pdf]

nature portfolio

Corresponding author(s): Peter Scheiffele
Last updated by author(s): 13/02/2024

Reporting Summary

Nature Portfolio wishes to improve the reproducibility of the work that we publish. This form provides structure for consistency and transparency in reporting. For further information on Nature Portfolio policies, see our Editorial Policies and the Editorial Policy Checklist.

Statistics

For all statistical analyses, confirm that the following items are present in the figure legend, table legend, main text, or Methods section.

n/a	Confirmed	
☐	☒	The exact sample size (*n*) for each experimental group/condition, given as a discrete number and unit of measurement
☐	☒	A statement on whether measurements were taken from distinct samples or whether the same sample was measured repeatedly
☐	☒	The statistical test(s) used AND whether they are one- or two-sided<br>*Only common tests should be described solely by name; describe more complex techniques in the Methods section.*
☐	☒	A description of all covariates tested
☐	☒	A description of any assumptions or corrections, such as tests of normality and adjustment for multiple comparisons
☐	☒	A full description of the statistical parameters including central tendency (e.g. means) or other basic estimates (e.g. regression coefficient) AND variation (e.g. standard deviation) or associated estimates of uncertainty (e.g. confidence intervals)
☒	☐	For null hypothesis testing, the test statistic (e.g. *F*, *t*, *r*) with confidence intervals, effect sizes, degrees of freedom and *P* value noted<br>*Give P values as exact values whenever suitable.*
☒	☐	For Bayesian analysis, information on the choice of priors and Markov chain Monte Carlo settings
☒	☐	For hierarchical and complex designs, identification of the appropriate level for tests and full reporting of outcomes
☒	☐	Estimates of effect sizes (e.g. Cohen's *d*, Pearson's *r*), indicating how they were calculated

Our web collection on statistics for biologists contains articles on many of the points above.

Software and code

Policy information about availability of computer code

Data collection	pClamp 11 (Molecular devices)<br>FLIR (Teledyne)<br>Zen (Zeiss)<br>SoftWorx 4.1.2 (API DeltaVision Core)<br>NextSeq 500 (Illumina)<br>NovaSeq 6000 (Illumina)

Data analysis	Igor Pro8 (WaveMetrics)<br>Neuromatic v3.0<br>Clampfit 10 (Molecular Devices)<br>Panther16.0 (pantherdb.org)<br>Hyugens Professional (Scientific Volume Imaging)<br>IMARIS 9.9.1 (Oxford Instruments)<br>ImageJ (NHI, 2.9.0/1.53t)<br>R Studio (Posit Software, PBC), Version 2022.12.0+353 (2022.12.0+353)<br>MACS2 (CZI EOSS) v2.1.3.3<br>limma/voom (Bioconductor.org), Limma. version 3.58.1<br>Homer (http://homer.ucsd.edu/homer/motif)<br>PANTHER 16 (http://pantherdb.org/)<br>Prism 9 (GraphPad)<br>ANY-MAZE v5.23 (Stoelting)

For manuscripts utilizing custom algorithms or software that are central to the research but not yet described in published literature, software must be made available to editors and reviewers. We strongly encourage code deposition in a community repository (e.g. GitHub). See the Nature Portfolio guidelines for submitting code & software for further information.

Data

Policy information about availability of data

All manuscripts must include a data availability statement. This statement should provide the following information, where applicable:

- Accession codes, unique identifiers, or web links for publicly available datasets
- A description of any restrictions on data availability
- For clinical datasets or third party data, please ensure that the statement adheres to our policy

ChIP-Seq and RNA-Seq data are aligned to mm10 mouse genome, and are deposited at GEO with accession numbers GSE255466, GSE255562, GSE255563 and GSE25587.

Human research participants

Policy information about studies involving human research participants and Sex and Gender in Research.

Reporting on sex and gender	N/A
Population characteristics	N/A
Recruitment	N/A
Ethics oversight	N/A

Note that full information on the approval of the study protocol must also be provided in the manuscript.

Field-specific reporting

Please select the one below that is the best fit for your research. If you are not sure, read the appropriate sections before making your selection.

☒ Life sciences ☐ Behavioural & social sciences ☐ Ecological, evolutionary & environmental sciences

For a reference copy of the document with all sections, see nature.com/documents/nr-reporting-summary-flat.pdf

Life sciences study design

All studies must disclose on these points even when the disclosure is negative.

Sample size	No statistical methods were used to predetermine the number of animals and cells. Suitable sample sizes were estimated based on previous published reports (Donato et al., Nature, 2013, Dehorter et al., Science, 2015, Mauger et al., Neuron, 2016, Xiao et al., Nature Communications, 2018, Hörnberg et al., Nature, 2020).
Data exclusions	Mice were excluded for CNO experiments when the expression of hM4Di virus was not sufficiently spread or if the mice had to be euthanized prematurely due to severe seizures. For patch clamp experiments, data was excluded if the recorded cells displayed >20% change in the series resistance during recordings.
Replication	All animal experiments were performed with animals from several litters. Each cohort showed similar phenotypes.<br>For immunostaining, western blot and FISH experiments, a minimum of 3 animals per genotype was used. For cell culture experiments, a minimum of 3 biological replicates were used and no attempts of experiments were excluded. N numbers are provided for each experiment in

the figure legends.

Randomization

Animals were randomly assigned to treatment groups at the time of viral injections. Cultures were also randomly assigned at the time of experiments.

Blinding

Experimenter was blinded to genotype for all experiments, and most analysis. For electrophysiological experiments, the experimenter was blinded for genotype. For EEG recordings, blinding for analysis was limited as the analysis was selective to identify the epileptiform brain activity. However, assessment for seizure detection and analysis were done by different experimenters, thereby enabling some blinding to the genotype.

Reporting for specific materials, systems and methods

We require information from authors about some types of materials, experimental systems and methods used in many studies. Here, indicate whether each material, system or method listed is relevant to your study. If you are not sure if a list item applies to your research, read the appropriate section before selecting a response.

Materials & experimental systems

n/a	Involved in the study
☐	☒ Antibodies
☐	☒ Eukaryotic cell lines
☒	☐ Palaeontology and archaeology
☐	☒ Animals and other organisms
☒	☐ Clinical data
☒	☐ Dual use research of concern

Methods

n/a	Involved in the study
☐	☒ ChIP-seq
☒	☐ Flow cytometry
☒	☐ MRI-based neuroimaging

Antibodies

Antibodies used

Primary antibodies:
rabbit Smad1, Cell Signaling, 6944, lot #6, 1to100 for ChIP and 1to1000 for WB
rabbit Smad5, Cell Signaling, 12534, lot #2, 1to100 for ChIP and 1to1000 for WB
rabbit-anti-phospho-SMAD1/5/9, Cell Signaling, 13820, lot #3, 1to800 for ICC and 1to1000 for WB
rabbit-anti-H3K27ac, Abcam 4729, lot #GR3231988-1, 1to1000
mouse-anti-BMPR2, BD Pharmingen, 612292, lot #7131991, 1to1000
rabbit-anti-Calnexin, stressGen, SPA-865, lot #11041924, 1to2000
rabbit-anti-HA, Cell Signaling, 3724, lot #10, 1to1000
rat-anti-GAPDH, Biolegend, 607902, lot #B259205, clone W17079A, 1to10000
mouse-anti-MAP2, Synaptic systems, 188011, lot #1-17, clone 198A5, 1to1000
mouse-anti-CamKII alpha, ThermoFisher, MA1-048, lot #TH269517, 1to800
mouse-anti-GAD67, Millipore, MAB5406, lot #53872310, clone #1G10.2, 1to500
rabbit-anti NeuN, Abcam ab177482, lot#1001571-1, 1to500
goat anti-Parvalbumin antibody, Swant, PVG214, RRID: AB_10000345, 1to5000
biotinylated WFA, Vector laboratories, B-1355-2, lot #SLBQ2585V, 1to500
Monoclonal mouse-anti-Synaptotagmin 2, Zebrafish International Resource Center, ZNP-1, RRID: AB_10013783, 1to1000
rabbit-anti-vGlut1 polyclonal purified antibody, Synaptic Systems, 135303, lot #4-90, 1to5000
mouse anti-GFP, Santa Cruz, sc-9996, 1to1000

Secondary antibodies
goat anti-rat-HRP, Jackson ImmunoResearch, 112-035-143, lot #152008, 1to10000
goat-anti-mouse-HRP, Jackson ImmunoResearch, 115-035-149, lot #120343, 1to10000
goat anti-rabbit-HRP, Jackson ImmunoResearch, 111-035-003, 1to10000
Cy3-conjugated donkey anti-mouse, Jackson ImmunoResearch, 715-165-151, lot #164090, 1to500
Cy3-conjugated donkey anti-rabbit, Jackson ImmunoResearch, 711-165-152, lot #160467, 1to500
Cy5-conjugated donkey anti goat, Jackson ImmunoResearch, 705-175-147, lot #818817, 1to500
Alexa405 goat anti-rabbit (Thermo Scientific #A-31556), 1to500
Alexa 488 conjugated donkey anti rabbit, ThermoFisher, R37118, 1to1000
Alexa 647 conjugated donkey anti mouse, Jackson ImmunoResearch, 715-605-151, lot #140647, 1to1000
Cy2-conjugated Streptavidin, Jackson ImmunoResearch, 016-220-084, 1to1000
Alexa 647 conjugated steptavidin, Thermo Fisher, A31556, lot # 1010119, 1to1000
Cy5-conjugated donkey-anti-rabbit, Jackson ImmunoResearch, 711-175-152, lot #161788, 1to500
Cy5 donkey anti-mouse, Jackson #715-175-511, Lot #165683, 1to500
Cy3-conjugated donkey-anti-guineapig, Jackson ImmunoResearch, 706-165-148, lot #154462, 1to500

Validation

Smad1/Smad5: WB: validated using various cell cell lines. ChIP: Chromatin immunoprecipitations were performed with cross-linked chromatin from MCF7 cells treated with Human BMP2 for one hour and either Smad1 Rabbit mAb or Normal Rabbit IgG (cell.signal.com)
H3K27ac: ChIP: Chromatin immunoprecipitations were performed with cross-linked chromatin from HeLa cells (abcam.com)
pSmad1/5/9: IHC: validated from HT-1080 cells, serum-starved (overnight; left) or serum-starved and treated with Human BMP2 #4697 (50 ng/ml, 30 min; right)
GAPDH: validated by western blotting using extracts from K562, PC3, HeLa, Molt-4, NTRA-2, NIH3T3, and UMR-106 cells

(biolegend.com)
Calnexin: validated by western blotting using extracts from MWM, Vero, 3T3, PC-12 and HeLa cells (enzolifesciences.com)
BMPR2: validated by western blotting using pulmonary artery smooth muscle cell extract (labome.com)
HA: validated by western blotting of extracts from HeLa cells, untransfected or transfected with either HA-FoxO4 or HA-Akt3 (cellsignal.com)
MAP2: validated for immunocytochemistry from primary rat hippocampal neurons (sysy.com)
anti CamKII alpha: validated for immunocytochemistry from primary mouse cortical cultures (thermofisher.com)
anti-GAD67: validated for immunohistochemistry from mouse and rat tissue (merckmillipore.com)
anti-NeuN: validated for immunohistochemistry from mouse and human tissue (abcam.com)
anti-Parvalbumin antibody: Validated for immunohistochemistry from parvalbumin knock-out mouse cortex and hippocampus tissues (swant.com)
anti-GFP antibody: validated by western blotting using extracts from COS cells, untransfected or transfected with GFP (scbt.com)
biotinylated WFA: validated for immunohistochemistry from octadon degu Alzheimer model (vectorlabs.com)
Monoclonal mouse-anti-Synaptotagmin 2: validated for immunohistochemistry in brain tissues (zfin.org)
rabbit-antivGlut1 polyclonal purified antibody: validated for immunohistochemistry by staining of hippocampus sections from mouse (sysy.com)

Eukaryotic cell lines

Policy information about cell lines and Sex and Gender in Research

Cell line source(s)	Primary cortical cells were prepared from the 16.5 days old embryos of RjOrl:SWISS mice (Janvier) mice or P0 pups of C57BL/6j mice. AAVpro HEK293T cell line was obtained from TakaraBio (#632273) and used at passage numbers from 8-20.
Authentication	Cell lines evaluated by observing their morphology and growth.
Mycoplasma contamination	Regular inspection of cell health and survival didn't show any indication for mycoplasma contamination.
Commonly misidentified lines (See ICLAC register)	no commonly misidentified lines were used in this study.

Animals and other research organisms

Policy information about studies involving animals; ARRIVE guidelines recommended for reporting animal research, and Sex and Gender in Research

Laboratory animals	Mice:<br>C57BL/6j, both males and females, P0 to adult (8-16 weeks)<br>RjOrl:SWISS mice (Janvier),male, female, E16.5
Wild animals	the study did not involve wild animals
Reporting on sex	Findings were obtained from both males and females. Sex was assigned by trained caretakers before toe marking for genotyping or at the weaning age. For in vitro experiments cells were isolated from embryos or P0 pups, sex information was not collected. For other experiments, matching number of animals for males and females were used.
Field-collected samples	the study did not involve field-collected samples
Ethics oversight	Basel Cantonal Veterinary Office Committees for Animal Experimentation

Note that full information on the approval of the study protocol must also be provided in the manuscript.

ChIP-seq

Data deposition

☒ Confirm that both raw and final processed data have been deposited in a public database such as GEO.

☒ Confirm that you have deposited or provided access to graph files (e.g. BED files) for the called peaks.

Data access links<br>*May remain private before publication.*	https://www.ncbi.nlm.nih.gov/geo/query/acc.cgi?acc=GSE255466<br>https://www.ncbi.nlm.nih.gov/geo/query/acc.cgi?acc=GSE255587
Files in database submission	smadsample1_R1.fastq.gz<br>smadsample1_R2.fastq.gz<br>smadsample2_R1.fastq.gz<br>smadsample2_R2.fastq.gz<br>smadsample3_R1.fastq.gz<br>smadsample3_R2.fastq.gz<br>smadsample4_R1.fastq.gz<br>smadsample4_R2.fastq.gz<br>input_cneg1_R1.fastq.gz<br>input_cneg1_R2.fastq.gz

input_cneg2_R1.fastq.gz
input_cneg2_R2.fastq.gz
input_cneg3_R1.fastq.gz
input_cneg3_R2.fastq.gz
input_cneg4_R1.fastq.gz
smad_vs_input_rep1_peaks.narrowPeak
smad_vs_input_rep2_peaks.narrowPeak
smad_vs_input_rep3_peaks.narrowPeak
smad_vs_input_rep4_peaks.narrowPeak
Bmp2_Input_rep1_S1_R1_001_MM_1.fastq.gz
Bmp2_Smad1_5_rep1_S2_R1_001_MM_1.fastq.gz
Bmp2_H3K27ac_rep1_S2_R1_001_MM_1.fastq.gz
NT_Input_rep1_S1_R1_001_MM_1.fastq.gz
NT_Smad1_5_rep1_S2_R1_001_MM_1.fastq.gz
NT_H3K27ac_rep1_S2_R1_001_MM_1.fastq.gz
Bmp2_Input_rep2_S1_R1_001_MM_1.fastq.gz
Bmp2_Smad1_5_rep2_S2_R1_001_MM_1.fastq.gz
Bmp2_H3K27ac_rep2_S2_R1_001_MM_1.fastq.gz
NT_Input_rep2_S1_R1_001_MM_1.fastq.gz
NT_Smad1_5_rep2_S2_R1_001_MM_1.fastq.gz
NT_H3K27ac_rep2_S2_R1_001_MM_1.fastq.gz
Bmp2_Input_rep3_S1_R1_001_MM_1.fastq.gz
Bmp2_Smad1_5_rep3_S2_R1_001_MM_1.fastq.gz
Bmp2_H3K27ac_rep3_S2_R1_001_MM_1.fastq.gz
NT_Input_rep3_S1_R1_001_MM_1.fastq.gz
NT_Smad1_5_rep3_S2_R1_001_MM_1.fastq.gz
NT_H3K27ac_rep3_S2_R1_001_MM_1.fastq.gz
Bmp2_Input_rep1_S1_R2_001_MM_1.fastq.gz
Bmp2_Smad1_5_rep1_S2_R2_001_MM_1.fastq.gz
Bmp2_H3K27ac_rep1_S2_R2_001_MM_1.fastq.gz
NT_Input_rep1_S1_R2_001_MM_1.fastq.gz
NT_Smad1_5_rep1_S2_R2_001_MM_1.fastq.gz
NT_H3K27ac_rep1_S2_R2_001_MM_1.fastq.gz
Bmp2_Input_rep2_S1_R2_001_MM_1.fastq.gz
Bmp2_Smad1_5_rep2_S2_R2_001_MM_1.fastq.gz
Bmp2_H3K27ac_rep2_S2_R2_001_MM_1.fastq.gz
NT_Input_rep2_S1_R2_001_MM_1.fastq.gz
NT_Smad1_5_rep2_S2_R2_001_MM_1.fastq.gz
NT_H3K27ac_rep2_S2_R2_001_MM_1.fastq.gz
Bmp2_Input_rep3_S1_R2_001_MM_1.fastq.gz
Bmp2_Smad1_5_rep3_S2_R2_001_MM_1.fastq.gz
Bmp2_H3K27ac_rep3_S2_R2_001_MM_1.fastq.gz
NT_Input_rep3_S1_R2_001_MM_1.fastq.gz
NT_Smad1_5_rep3_S2_R2_001_MM_1.fastq.gz
NT_H3K27ac_rep3_S2_R2_001_MM_1.fastq.gz
Bmp2_Input_1.bw
Bmp2_Smad1_5_1.bw
Bmp2_H3K27ac_1.bw
NT_Input_1.bw
NT_Smad1_5_1.bw
NT_H3K27ac_1.bw
Bmp2_Input_2.bw
Bmp2_Smad1_5_2.bw
Bmp2_H3K27ac_2.bw
NT_Input_2.bw
NT_Smad1_5_2.bw
NT_H3K27ac_2.bw
Bmp2_Input_3.bw
Bmp2_Smad1_5_3.bw
Bmp2_H3K27ac_3.bw
NT_Input_3.bw
NT_Smad1_5_3.bw
NT_H3K27ac_3.bw

Genome browser session
(e.g. UCSC)

N/A

Methodology

Replicates

3 biological replicates that were generated from independent litters were used for in vitro ChIP-seq experiments. 4 biological replicates (2 males and 2 females) were used for in vivo ChiP-seq experiments.

Sequencing depth

The sequencing was performed paired end 41 bases yielded around 50±2 millions pass filter reads for in vitro and 19-32 millions pass filter reads for in vivo samples..

Antibodies	rabbit anti-Smad1/Smad5 (Cell Signaling #6944 and #12534) and rabbit anti-H3K27ac (Abcam 4729) antibodies were used.
Peak calling parameters	Peaks were called for each ChIP replicate against a matched input using the MACS2 callpeak function with the default options.
Data quality	Peaks existed in at least 2 out of 3 replicates for each group were considered which yielded in total 896 peaks. For comparisons between control and Bmp2 treated conditions, peaks that had at least 1.4 fold change and less then 0.05 adjusted p-value were considered as significant. For in vivo ChiP-seq, peaks existed in at least 3 out of 4 replicates were considered which yielded 239 peaks.
Software	MACS2 and R softwares were used by using default parameters for analysis.
